# Effects of continuous ketamine infusion on hemodynamics and mortality in critically ill children

**DOI:** 10.1371/journal.pone.0224035

**Published:** 2019-10-18

**Authors:** Sojin Park, Ah Young Choi, Esther Park, Hyo Jung Park, Jaehyun Lee, Hukyoung Lee, JeongMee Kim, Joongbum Cho

**Affiliations:** 1 Department of Pharmaceutical Services, Samsung Medical Center, Seoul, Republic of Korea; 2 Department of Critical Care Medicine, Samsung Medical Center, Sungkyunkwan University School of Medicine, Seoul, Republic of Korea; Wayne State University, UNITED STATES

## Abstract

We investigated the hemodynamic and mortality effects of continuous ketamine infusion in critically ill pediatric patients. We conducted a retrospective cohort study in a tertiary pediatric intensive care unit (PICU). Patients who used continuous sedative from 2015 to 2017 for 24 hours or more were included. We compared blood pressure, heart and respiratory rates, vasogenic medications, and sedation and pain scores for 12 hours before and after initiation of continuous ketamine. The mortality rates for continuous ketamine and Non-ketamine groups were compared by multivariate logistic regression. A total of 240 patients used continuous sedation, and 82 used continuous ketamine. The median infusion rate of ketamine was 8.1 mcg/kg/min, and the median duration was 6 days. Heart rates (138 vs. 135 beat/minute, *P* = .033) and respiratory rates (31 vs. 25 respiration/minute, *P* = .001) decreased, but blood pressure (99.9 vs. 101.1 mm Hg, *P* = .124) and vasogenic medications did not change after ketamine infusion. Continuous ketamine was not a significant risk factor for mortality (hazard ratio 1.352, confidence interval 0.458–3.996). Continous ketamine could be used in PICU without hemodynamic instability. Further studies in randomized controlled design about the effects of continuous ketamine infusion on hemodynamic changes, sedation, and mortality are required.

## Introduction

Pediatric patients in intensive care units (ICUs) are frequently treated with sedatives and analgesics because critical care is a stressful, painful experience [1]. One study reported that 25% of patients with stressful ICU experiences showed symptoms of post-traumatic stress disorder 4 years later [2]. The proportion of mechanically ventilated patients prescribed continuous intravenous sedation has increased over time [3]. However, based on concerns about benzodiazepine-induced delirium and opioid tolerance, healthcare providers are seeking alternative or additive agents.

Ketamine is a noncompetitive antagonist of N-methyl-D-aspartate (NMDA) receptor that has analgesic and antihyperalgesic properties. It is an ideal anesthetic because it dose-dependently produces analgesia, amnesia, unconsciousness, and akinesia [4]. Critically ill patients experience pain more readily than healthy people, and up to half of critically ill patients experience pain while at rest, without noxious stimuli [5,6]. Due to its characteristics, ketamine is considered an appropriate additive drug for ICU analgosedation. It has catecholamine-releasing effects that could favorably affect cardiovascular parameters in a shock state. However, studies describing the hemodynamic effects of prolonged ketamine infusion are limited in pediatric patients. Only case reports and case series on long-term, continuous ketamine infusion are available, along with studies that evaluated short infusion durations of 2–3 hours [7–11]. A recent analysis of 72 hours of continuous ketamine infusion for analgosedation in the ICU was based solely on data from an adult population [12]. In this study, we investigated the hemodynamic and sedative effects of continuous ketamine infusion and determined if continuous ketamine infusion affected mortality rates of critically ill pediatric patients.

## Methods

### Study setting and patients

In this retrospective cohort study, we screened and reviewed all consecutive admissions from January 2015 to December 2017 to a pediatric intensive care unit (PICU) of a tertiary hospital. The PICU was a 15-bed, combined medical-surgical unit. Patients with congenital heart disease or organ transplantation were admitted to other dedicated units immediately post operation. In the PICU under study, opioid-based analgosedatives were used as an initial sedative for most mechanically ventilated patients according to analgesia-first sedation suggested in the guideline for pain, agitation, and delirium management [13]. Continuous ketamine was used mostly as an add on sedative for further sedation when the first line medications failed to sedate adequately. We included patients who used any continuous sedative medication for 24 hours or more during the study period. We excluded patients who used sedatives less than 24 hours since our study's aim was to observe the effect of prolonged continuous ketamine use.

### Data collection

Data on eligible patients and the use of sedatives were supplied by a licensed medical records officer from the electronic medical record (EMR). Data on continuous medication which were recorded in EMR sheets included medication name, infusion rate and dose, start and finish times, change of dose, and time of change. Among the PICU-admission cohort, records for patients with a history of continuous medication or with sedative orders for more than 2 consecutive days were reviewed. Patients’ charts and prescriptions were reviewed by researchers who were pediatric intensive care physicians and pharmacists. Patients who had ketamine as a continuous sedative were put in a Ketamine group, and patients who had any sedative other than ketamine as a continuous sedative were put in a Non-ketamine group.

Medication changes to control blood pressure were reviewed by ICU pharmacists for the periods 12 hours before and after the start of continuous ketamine medication. Data were collected on addition, deletion, or dose change in intravenous or oral antihypertensives or vasopressors (dopamine, dobutamine, epinephrine, norepinephrine, vasopressor, milrinone) to evaluate hemodynamic changes which required treatment modification. Data on changes in sedative medications were collected 12 hours before and after the start of continuous ketamine infusion. Vasoactive-inotropic score (VIS) was calculated to compare the dose of vasopressor [14], and antihypertensive therapeutic intensity score (TIS) was calculated to compare the dose of antihypertensives [15].

Vital signs were recorded in EMRs hourly except in unstable and critical situations. We collected systolic, diastolic, and mean blood pressure values; heart rates; and respiratory rates 12 hours before and after the start of continuous ketamine infusion. For each, we used the mean value over 12 hours for comparisons. For sedation and pain evaluation, the Richmond Agitation-Sedation Scale (RASS) and Face Pain Scale (FPS) or Face-Leg-Activity-Cry-Consolability (FLACC) scores were measured at least every 8 hours and at other frequencies according to clinical changes. We collected the RASS and FPS or FLACC scores 12 hours before and after the start of continuous ketamine infusion and used the mean values over 12 hours for comparison.

### Statistical analysis

Descriptive statistics were used for age, sex, underlying disease, use of vasopressors or mechanical ventilation, duration of mechanical ventilation, pre-ICU hospital stay, ICU and hospital lengths of stay, predicted mortality, and actual mortality. Parameters were compared between the Ketamine and Non-ketamine groups by Mann-Whitney test. Paired tests were used to compare parameters between pre-ketamine infusion and post-ketamine infusion. We compared systolic, diastolic, and mean blood pressures; heart rates; and respiratory rates by paired t-test; and antihypertensive and vasopressor use by McNemer test. We compared RASS, pain-control scores, and the doses of other sedatives between pre and post ketamine infusion by Wilcoxon signed-rank test. To evaluate risk factors associated with mortality, we conducted multivariate linear regression tests using age; sex; use of mechanical ventilation or vasopressor; use of continuous ketamine, fentanyl, benzodiazepine, or dexmedetomidine; pre-ICU hospital days; predicted mortality at admission; and underlying oncologic or cardiac disease.

### Ethics statement

The study protocol was reviewed and approved by the Institutional Review Board of Samsung Medical Center (IRB No. 2018-02-010). The Institutional Review Board waived the need for informed consent for this study.

## Results

**Fig 1:**
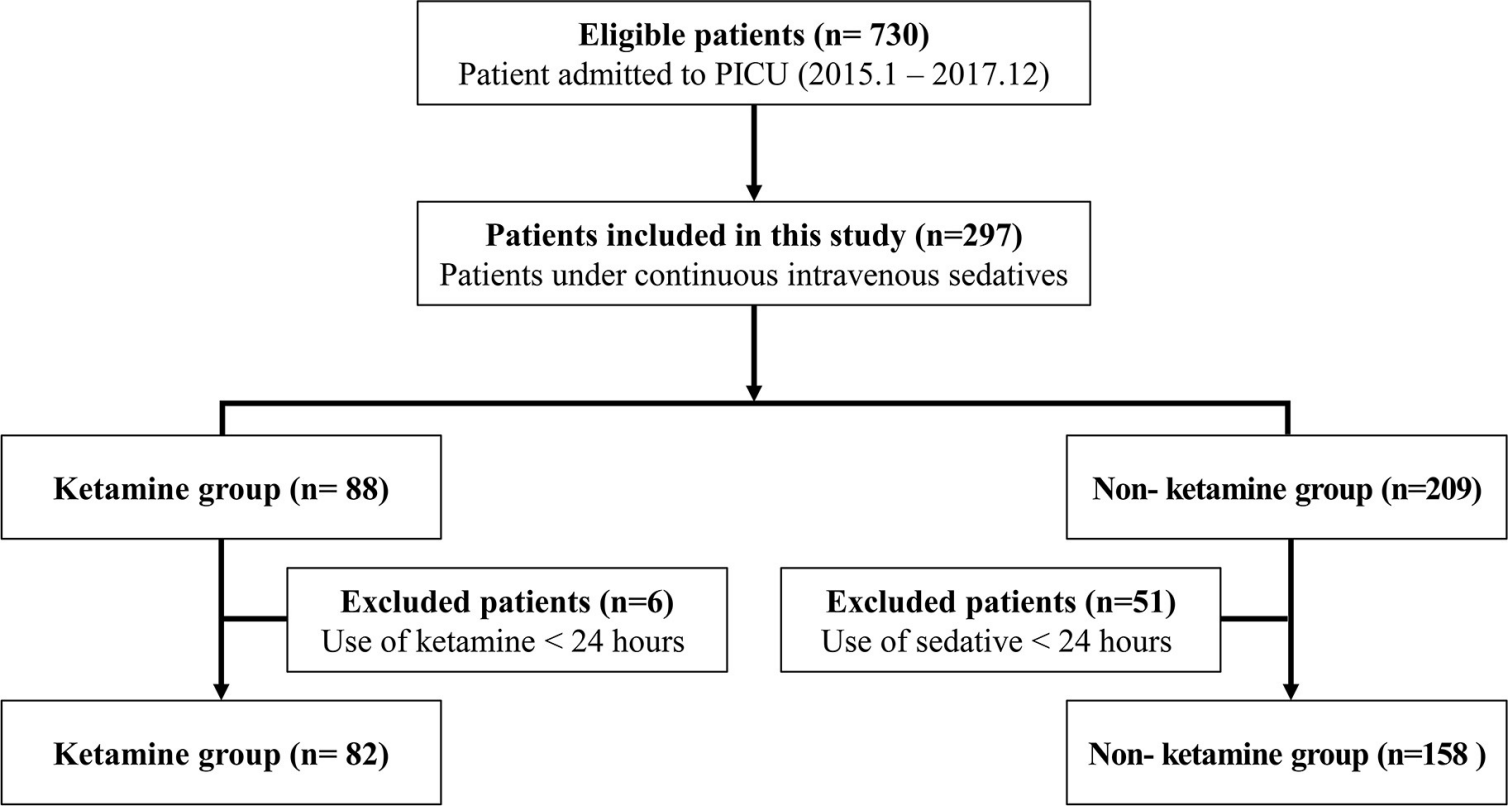
Flow diagram of patient selection with inclusion and exclusion criteria. PICU, pediatric intensive care unit.

A total of 730 patients were admitted to the PICU, and 297 received at least one sedative agent. After excluding 6 patients who were administered continuous ketamine for less than 24 hours, the final Ketamine group was 82 patients. After excluding 51 patients who were administered any continuous sedatives for less than 24 hours, the Non-ketamine group was 158 patients (Fig 1). Patient age was higher in the Ketamine than the Non-ketamine group (2.1 vs. 1.1 years, *P* = .008). Although underlying disease was not significantly different between groups, oncologic disease was more common in the Ketamine group (32.9% vs. 15.8%), and cardiac disease was more common in the Non-ketamine group (19.5% vs. 32.3%). Application of mechanical ventilation was similar between the Ketamine and Non-ketamine groups (89.0% vs. 91.9%, *P* = .646). Dexmedetomidine was more commonly used in the Ketamine group (35.4% vs. 19.6%, *P* = .011), and the number of sedatives used was higher in the Ketamine group (3 vs. 1 sedative, *P* < .001). The Ketamine group, compared to the Non-ketamine group, had longer pre-ICU hospital stay (2 vs. 0 days, *P* = .003), duration of mechanical ventilation (17.0 vs. 7.5 days, *P* < .001), ICU length of stay (22.0 vs. 12.0 days, *P* < .001), and hospital length of stay (52.2 vs. 28.9 days, *P* < .001). The mean predicted mortality of total study population was 15.4%, and actual mortality was 18.7% 945/240). The standard mortality ratio was 1.21 (95% confidence interval: 0.80–1.62). Median predicted mortality derived from severity score at admission was higher in the Ketamine group (7.1 vs. 4.0%, *P* = .041), as was crude mortality (31.7 vs. 12.0%, *P* < .001) (Table 1). The standardized mortality rate of the Ketamine group was 1.93 (95% CI: 1.26–2.60), and the standardized mortality rate of the Non-ketamine group was 0.80 (95% CI: 0.34–1.25).

**Table 1:** Characteristics and treatment outcomes of patients with continuous sedation.

Variables	Ketamine group (n = 82)	Non-ketamine group (n = 158)	*P* Value
Age, median year (IQR)	2.1 (0.7, 5.9)	1.1 (0.3, 5.0)	.008
Male, n (%)	42 (51.2)	88 (55.7)	.585
Underlying disease, n (%)			.067
Oncology	27 (32.9)	25 (15.8)	
Respiratory disease	24 (29.3)	40 (25.3)	
Cardiac disease	16 (19.5)	51 (32.3)	
GI/hepatic disease	4 (4.9)	10 (6.3)	
Other disease	11 (13.4)	32 (20.2)	
Treatment, n (%)			
Vasopressors	63 (76.8)	101 (63.9)	.057
Mechanical ventilator	73 (89.0)	144 (91.1)	.646
Other sedatives			
Fentanyl	64 (78.0)	120 (75.9)	.750
Midazolam	52 (63.4)	97 (61.4)	.781
Dexmedetomidine	29 (35.4)	31 (19.6)	.011
Number of used sedatives	3 (2–3)	1 (1–2)	.000
Pre-ICU hospital stay	2 (0–21)	0 (0–8.2)	.003
Mechanical ventilation day	17.0 (8.0, 43.0)	7.5 (3.0, 12.0)	.000
ICU length of stay	22.0 (10.0, 48.0)	12.0 (6.0, 21.2)	.000
Hospital length of stay	52.2 (25.2, 89.5)	28.9 (16.7, 57.5)	.000
Predicted mortality %,			
median (IQR)	7.14 (2.60, 17.50)	4.00 (1.74, 12.91)	.041
mean (SD)	16.4 (23.4)	14.9 (25.0)	
Mortality, n (%)	26 (31.7)	19 (12.0)	.000

IQR, interquartile range; ICU, intensive care unit; SD, standard deviation.

Mechanical ventilation day and length of stay are presented as median days (interquartile range).

Median ketamine infusion rate was 8.1 mcg/kg/min (IQR, 5.0–12.8 mcg/kg/min), and median duration of continuous infusion was 6 days (IQR, 2.7–17 days) in Ketamine group. Among 82 patients of Ketamine group, ketamine was the first sedative drug for 24 (29.2%), the second for 27 (32.9%), the third for 29 (35.4%) and the fourth for 2 (2.4%). In the Ketamine group, 10 patients (12.2%) received only ketamine as a continuous sedative.

Comparisons of hemodynamic parameters for pre and post 12 hours of continuous ketamine use are presented in Table 2. Heart rate (138 vs. 135 beat/minute, *P* = .033) and respiratory rate (31 vs. 25 breath/minute, *P* = .001) decreased after initiation of continuous ketamine infusion. Blood pressure (BP) did not change significantly, but VIS (0 vs. 3.4, *P* < .001) slightly increased after continuous ketamine infusion (Table 2). RASS sedation score decreased (0 vs. -1, *P* = 0.06) and fentanyl dose increased (3.0 vs. 4.0 mcg/kg/min, *P* < .001) after continuous ketamine infusion (Table 3).

**Table 2:** Comparison of vital signs and medications for 12 hours pre and post continuous ketamine use.

Vital signs and medications	Pre-ketamine	Post-Ketamine	*P* Value
Blood pressure, mmHg			
Systolic BP (SD)	99.9 (±16.6)	101.1 (±15.1)	.124
Diastolic BP (SD)	57.3 (±15.2)	57.5 (±12.5)	.851
Mean BP (SD)	67.7 (±15.3)	69.6 (±12.9)	.181
Heart rate, beat/min (SD)	138 (±26)	135 (±23)	.033
Respiratory rate, respiration/min (SD)	31 (±16)	25 (±12)	.001
Blood pressure control (n = 82)			
Antihypertensive use, n (%)*	9 (11.0)	13 (15.9)	.125
Antihypertensive TIS	0.3 (0.1, 0.7)	0.2 (0.1, 0.5)	.139
Vasopressor use, n (%)*	23 (28.0)	29 (35.4)	.31
Vasoactive-inotropic score	0 (0.0, 6.3)	3.4 (0.1, 22.1)	< .001
Blood pressure control of non-hypotensive patients (n = 60)			
Antihypertensive use, n (%)*	6 (7.3)	9 (11.0)	.250
Antihypertensive TIS	0.4 (0.2, 0.8)	0.3 (0.1, 0.7)	.462
Vasopressor use, n (%)*	7 (8.5)	8 (9.8)	1.000
Vasoactive-inotropic score	0 (0.0, 2.3)	0 (0.0, 4.9)	< .001

BP: blood pressure; SD, standard deviation; CIV, continuous intravenous infusion; TIS, therapeutic index score.

*n (%) indicates the number of patients (percentage of each group) who use antihypertensives or vasopressors.

**Table 3:** Comparison of sedative effects and other sedative medication dosage changes for 12 hours pre and post continuous ketamine use.

Sedation/pain control parameter	Pre-ketamine	Post-ketamine	*P* Value
RASS average, median (IQR)	0 (-3, 2)	-1 (1, -5)	.06
Pain control score, median (IQR)	0 (0, 2)	0 (0, 1)	.10
Another sedative dose, median (IQR)			
Fentanyl, mcg/kg/min	3.00 (1.00, 5.00)	4.00 (2.10, 6.00)	.000
Midazolam, mg/kg/hr	0.13 (0.07, 0.20)	0.18 (0.08, 0.20)	.340
Dexmedetomidine, mcg/kg/hr	0.30 (0.07, 0.50)	0.37 (0.00, 0.54)	.754

RASS, Richmond Agitation-Sedation Scale; IQR, interquartile range.

In multivariate analysis of risk factors associated with mortality, use of continuous ketamine infusion was not a significant risk factor for mortality increase (hazard ratio [HR] 1.352, confidence interval [CI] 0.458–3.996) (Table 4). Sex and use of mechanical ventilation, vasopressor, fentanyl, benzodiazepine, or dexmedetomidine were included in multivariate analysis, but none showed statistical significance. Age (HR 1.089, CI 1.005–1.182), pre-ICU hospital stay (HR 1.006, CI 1.001–1.011), predicted mortality (HR 1.028, CI1.011–1.045), underlying oncologic disease (HR 4.119, CI 1.531, 11.085), and underlying cardiac disease (HR 0.212, CI 0.053–0.845) were significant risk factors associated with mortality.

**Table 4:** Multivariate linear regression for risk factors associated with mortality in continuously sedated patients.

Risk factors	HR (95% CI)	*P* Value
Ketamine use	1.352 (0.458, 3.996)	.585
Admission age (year)	1.089 (1.005, 1.182)	.038
Pre-ICU hospital stay (day)	1.006 (1.001, 1.011)	.012
Predicted mortality (%)	1.028 (1.011, 1.045)	.001
Underlying disease		
Oncologic disease	4.119 (1.531, 11.085)	.005
Cardiac disease	0.212 (0.053, 0.845)	.028

HR, hazard ratio; CI, confidence interval.

Multivariate linear regression included parameters of age; sex; use of mechanical ventilation, vasopressor; continuous ketamine, fentanyl, benzodiazepine, or dexmedetomidine; pre-ICU hospital days; predicted mortality at admission; and underlying oncologic disease.

## Discussion

In this study, we showed that continuous ketamine infusion with or without other sedatives did not change blood pressure significantly with an increase in median VIS 3.4. After starting continuous ketamine, patients were more sedated, although opioid administration also increased. We did not find that continuous ketamine infusion significantly affected mortality rate in the severity-adjusted analysis.

Ketamine administration is reported to increase catecholamine concentration in serum and cerebrospinal fluid and to increase sympathetic activity and blood pressure [16–18]. Ketamine is considered a rational choice for rapid sequence induction in hemodynamically compromised patients [19,20]. However, ketamine without sympathetic stimulation depresses cardiac contractility in isolated animal hearts [21,22]. Ketamine also produces negative inotropic effects in patients with chronic catecholamine depletion or ischemic heart disease [23,24]. However, data are lacking on the effects of ketamine on blood pressure in critically ill patients [9]. Earlier studies of ketamine effects on hemodynamic parameters were conducted on patients spontaneously breathing room air [25]. In ICU settings, case series and studies with small numbers of patients receiving ketamine infusion found no significant cardiovascular compromise [26–28]. However, these studies had no comparison of parameters before and after ketamine initiation. Our study compared systolic, diastolic, and mean blood pressures; these means did not change after ketamine infusion. To identify the number of patients who had started or stopped medication newly to preserve blood pressure, we evaluated the number of patients who use vasopressor or antihypertensive. There were no significant changes in patients numbers after initiation of ketamine. Vasopressor dose was elevated after initiation of ketamine infusion. It could be a result of a patient’s worsening condition since many sedatives start when a patient begins to aggravate. The change of VIS could be a result of the hemodynamic effect of ketamine, and it lowered blood pressure. However, VIS was not correlated with increased doses of continuous ketamine (S1 Fig), so we think VIS increased because of patients' condition (Table 2). Even though ketamine decreased blood pressure, we think the change of 3 microgram/kg/min of dopamine or dobutamine (equivalent to median changes of VIS) is tolerable for stabilization of blood pressure. Heart and respiratory rates decreased in our results, even though ketamine preserves heart and respiratory rate [19,29,30] or increases heart rate [31]. We presume that high doses of continuous ketamine in our study might have contributed to heart and respiratory rate decreases. In this study, we could not evaluate the sole effect and dose-dependent effect of ketamine on hemodynamic parameters. Although dose-response on hemodynamic parameter was not definite in our studying dose (S1 Fig), the hemodynamic effect of high anesthetic dose or low sub-dissociative dose for pain control should be studied further.

Our results showed a significant decrease in RASS score after ketamine infusion. This result might have been caused by an additional sedative effect of ketamine or by increased fentanyl dose. Ketamine was given to patients who required deeper sedation. Therefore, we hypothesize that an increase in mean fentanyl dose was not associated with ketamine infusion but with patient condition. In a previous study of 36 critically ill adult patients, the proportion of time that patients were at their goal RASS was not significantly different for 72 hours before or after ketamine initiation (median 83.3% vs. 83.3%, *P* = .416), and opioid use decreased after ketamine (0.81 mg/kg/hr) initiation [12]. In a randomized control study, low-dose ketamine (0.2 mg/kg/hr) infusion was associated with decreased delirium but not with decreased opiate consumption in patients undergoing mechanical ventilation [32]. Ketamine mainly acts by blocking NMDA receptor, but there are interactions with opioid receptors. The effects of ketamine on opioid receptor might differ with ketamine concentration according to some laboratory findings [33,34]. However, further studies about the clinical effect of ketamine on the opioid dose are required. Pain-control scores did not significantly change (0 vs. 0, *P* = .10) after continuous ketamine in our study since pain scores of the patients were already low before continuous ketamine.

Ketamine has possible benefits for survival in ICU patients. Decreased mortality and a significant reduction in tumor necrosis factor-α and interleukin-6 were reported in a septic rat model [35]. In another animal study, ketamine inhibited albumin extravasation in a chemical peritonitis model [36]. In addition to anti-inflammatory effects, ketamine might have neuroprotective effects in ICU patients [37]. However, previous studies did not analyze the effects of continuous ketamine infusion on mortality in ICU patients [38]. In our study, mortality was higher in the Ketamine group in unadjusted analysis.

We hypothesized the high mortality of Ketamine group did not come from ketamine itself but came from the different clinical features such as progressed disease conditions after admission, poor treatment response, or opioid/benzodiazepine tolerance after prolonged use. We consider the practice pattern of adding ketamine after other medications fail for adequate sedation made this difference between the two groups. In this study, standardized mortality ratio adjusted by severity scoring was higher in the Ketamine group (1.93) than Non-ketamine group (0.80). Since the severity scoring reflects only the initial condition at admission, we adjusted more confounding factors, including the proportion of oncological disease numbers of sedatives used, and pre-ICU hospital days. In adjusted analyses, differences in mortality between the two groups were not statistically significant. In a meta-analysis, all-cause mortality with ketamine infusion in combination with midazolam was not different from the use of propofol, benzodiazepine, or alpha agonist [39]. We did not adjust for length of mechanical ventilation day, ICU stay, or hospital stay since those variables may reflect patient severity but also could be the effect of sedative itself including continuous ketamine sedation. Therefore, further studies in the randomized controlled design are required to determine the effect of continuous ketamine on mortality in ICU patients.

Our study has some limitations. First, this was a single-center study. We recruited as many feasible cases as possible, but we found that our study showed a 65% power with a two-sided *P* value of 0.05 for mortality outcome. Second, there are many differences between the two groups. We could not adjust for all mortality risk factors in the Ketamine group due to the retrospective nature of the study. However, we think that the mortality risk for continuous ketamine infusion might decrease if we adjusted for more risk factors when we consider the high severity of the Ketamine group. Third, adverse drug reactions such as salivation or hallucination before and after continuous ketamine infusion were not evaluated due to unsatisfactory records. Forth, we could not evaluate the dose-response of ketamine on the clinical parameters and mortality.

Despite the limitations, our study had the largest number of patients who used continuous ketamine among reported studies. This was the first study to show changes in vital signs after initiation of continuous ketamine infusion and compare mortality with continuous ketamine sedation in PICU patients.

## Conclusion

Continuous ketamine infusion could be used without hemodynamic instability in PICU patients. Further studies in randomized controlled design about the effects of continuous ketamine infusion on hemodynamic changes, sedation, and mortality are required.

## Supporting information

S1 Fig Scatter plots of hemodynamic changes after continuous ketamine infusion and the mean dose of continuous ketamine infusion. CC: correlation coefficient.
(DOCX)

## Abbreviations

PICU: Pediatric intensive care unit
ICU: intensive care unit
NMDA: N-methyl-D-aspartate
EMR: electronic medical record
RASS: Richmond Agitation-Sedation Scale
FPS: Face Pain Scale
FLACC: Face-Leg-Activity-Cry-Consolability
BP: blood pressure
HR: hazard ratio
CI: confidence interval
VIS: vasoactive-inotropic score
TIS: therapeutic intensity score
