# Supplementary material for: Effects of continuous ketamine infusion on hemodynamics and mortality in critically ill children
Source: PLoS One. 2019 Oct 18;14(10):e0224035. doi: 10.1371/journal.pone.0224035 (PMC6799949; doi:10.1371/journal.pone.0224035)
Supplement: S1 Fig — CC: correlation coefficient. (DOCX) [file pone.0224035.s001.docx]

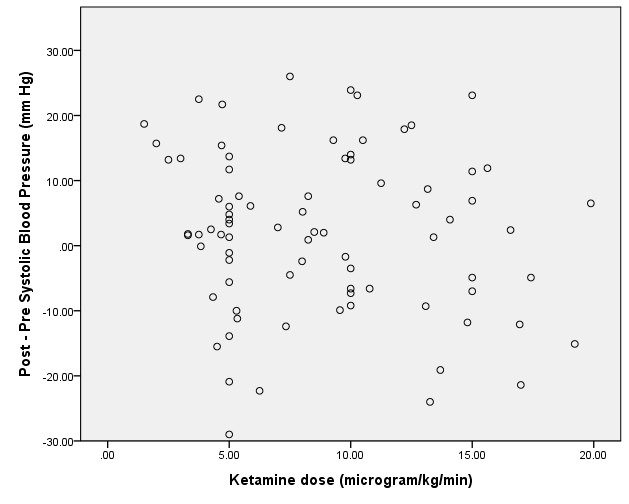

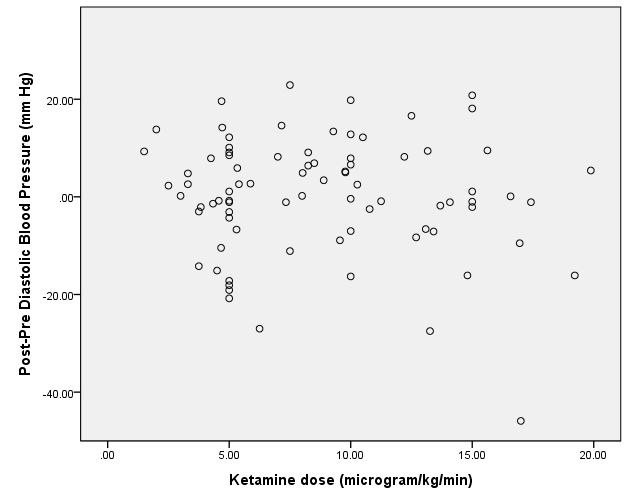


CC: -0.117 (P=0.297) CC: -0.041 (P=0.713)


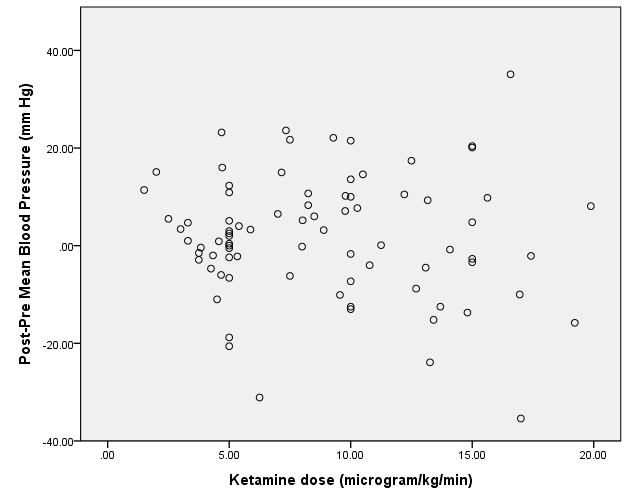

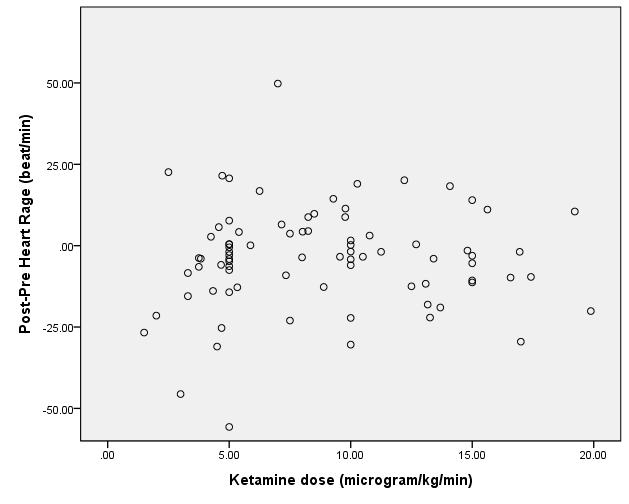


CC: -0.074 (P=0.512) CC: 0.065 (P=0.562)


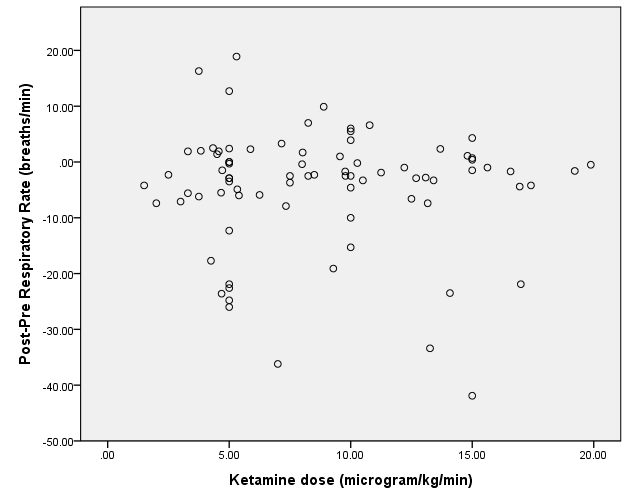

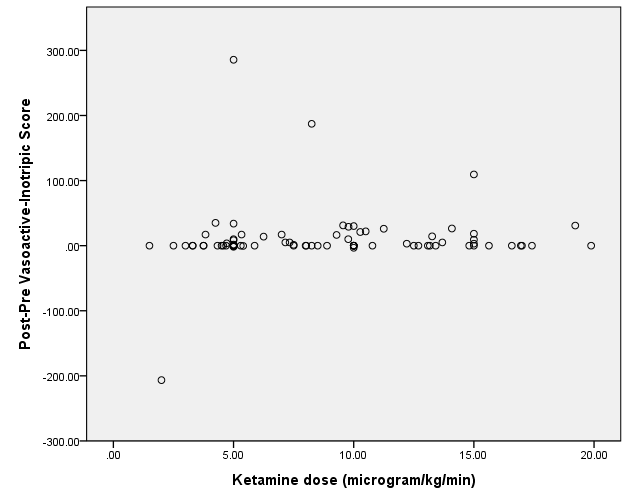


CC: 0.033 (P=0.771) CC: 0.177 (P=0.112)

Supplement Figure 1. Scatter plots of Hemodynamic changes after continuous ketamine infusion and the dose of continuous ketamine infusion. CC: correlation coefficient
